# Vorhersage des postoperativen Sprachverstehens mit dem transkutanen teilimplantierbaren Knochenleitungshörsystem Osia®

**DOI:** 10.1007/s00106-023-01336-4

**Published:** 2023-08-17

**Authors:** Susan Arndt, Thomas Wesarg, Antje Aschendorff, Iva Speck, Thomas Hocke, Till Fabian Jakob, Ann-Kathrin Rauch

**Affiliations:** 1 Klinik für Hals‑, Nasen- und Ohrenheilkunde, Universitätsklinikum Freiburg, Medizinische Fakultät, Albert-Ludwigs-Universität Freiburg, Killianstr. 5, 79106 Freiburg, Deutschland; 2 Cochlear Deutschland GmbH & Co KG, Mailänder Straße 4 a, 30539 Hannover, Deutschland

**Keywords:** Schallleitungsschwerhörigkeit, Kombinierte Schwerhörigkeit, Aktives transkutanes Knochenleitungssystem, Sprachverstehen, Knochenverankertes Hörsystem, Conductive hearing loss, Mixed hearing loss, Active transcutaneous bone conduction system, Speech comprehension, Bone-anchored hearing system

## Abstract

**Hintergrund:**

Das aktive transkutane, teilimplantierbare, osseointegrierte Knochenleitungssystem Cochlear™ Osia® (Fa. Cochlear, Sydney, Australien) ist seit April 2021 im deutschsprachigen Raum zugelassen. Das Osia ist für Patienten mit Schallleitungs- (SL-SH) oder kombinierter Schwerhörigkeit (komb-SH) mit einem mittleren Knochenleitungshörverlust von maximal 55 dB HL oder bei einseitiger Taubheit („single-sided deafness“, SSD) indiziert.

**Ziel der Arbeit:**

Ziel dieser retrospektiven Untersuchung war es, die Prädiktion des postoperativen Sprachverstehens mit Osia zu untersuchen sowie die Ergebnisse des Sprachverstehens von Patienten mit komb-SH und einem geringen Dynamikbereich von weniger als 30 dB nach erfolgter Osia-Versorgung zu evaluieren.

**Material und Methoden:**

Zwischen 2017 and 2022 wurden 29 erwachsene Patienten mit dem Osia versorgt, davon 10 Patienten (11 Ohren) mit SL-SH und 19 Patienten (25 Ohren) mit komb-SH. Die Patienten mit komb-SH wurden in 2 Gruppen aufgeteilt: komb-SH‑I mit über 4 Frequenzen gemittelter Hörschwelle in Knochenleitung („four-frequency pure-tone average“,(PTA4-KL)) ≥ 20 dB HL und < 40 dB HL (*n* = 15 Patienten, 20 Ohren) vs. komb-SH-II mit PTA4-KL ≥ 40 dB HL (*n* = 4 Patienten, 5 Ohren). Alle Patienten testeten präoperativ ein Knochenleitungsgerät am Softband. Präoperativ wurde das Sprachverstehen im Freiburger Einsilbertest unversorgt und mit Testsystem erfasst. Das maximale Einsilberverstehen (mEV) unversorgt und das Einsilberverstehen (EV) mit Testsystem bei 65 dB SPL wurde mit dem postoperativ erreichten EV mit Osia bei 65 dB SPL korreliert.

**Ergebnisse:**

Die präoperative Vorhersage für das postoperative Ergebnis mit Osia war anhand des mEV mit höherer Varianzaufklärung als mit dem EV bei 65 dB SPL mit KL-Testgerät am Softband möglich. Das postoperative EV war am besten für die Patienten mit SL-SH und am schlechtesten für Patienten mit komb-SH mit einer PTA4-KL ≥ 40 dB HL vorhersagbar. Die Ergebnisse mit dem Testgerät am Softband zeigen eher das minimal erreichbare Ergebnis und das mEV eher das realistisch zu erreichende Ergebnis mit Osia.

**Schlussfolgerung:**

Das Osia kann für die Versorgung von SL-SH und komb-SH unter Beachtung der Indikationsgrenzen eingesetzt werden. Auch die mittlere präoperative Knochenleitungshörschwelle liefert näherungsweise eine Abschätzung des postoperativen EV mit Osia, für das die genaueste Vorhersage anhand des präoperativen mEV erzielt wird. Die Vorhersagegenauigkeit reduziert sich ab einer PTA4-KL von ≥ 40 dB.

## Abstract (en)

**Background:**

The active transcutaneous, partially implantable osseointegrated bone conduction system Cochlear™ Osia® (Cochlear, Sydney, Australia) has been approved for use in German-speaking countries since April 2021. The Osia is indicated for patients with conductive (CHL) or mixed hearing loss (MHL) with an average bone conduction (BC) hearing loss of 55 dB or less, or with single-sided deafness (SSD).

**Objectives:**

The aim of this retrospective study was to investigate the prediction of postoperative speech recognition with Osia and to evaluate the speech recognition of patients with MHL and an aided dynamic range of less than 30 dB with Osia.

**Materials and methods:**

Between 2017 and 2022, 29 adult patients were fitted with the Osia, 10 patients (11 ears) with CHL and 19 patients (21 ears) with MHL. MHL was subdivided into two groups: MHL‑I with four-frequency pure-tone average in BC (BC-4PTA) ≥ 20 dB HL and < 40 dB HL (*n* = 15 patients; 20 ears) vs. MHL-II with BC-4PTA ≥ 40 dB HL (*n* = 4 patients; 5 ears). All patients tested a bone conduction hearing device on a softband preoperatively. Speech intelligibility in quiet was assessed preoperatively using the Freiburg monosyllabic test unaided and with the test system and postoperatively with Osia. The maximum monosyllabic score (mEV) unaided and the monosyllabic score with the test system at 65 dB SPL were correlated with the postoperative monosyllabic score with Osia at 65 dB SPL.

**Results:**

Preoperative prediction of postoperative outcome with Osia was better using the mEV than the EV at 65 dB SPL with the test device on the softband. Postoperative EV was most predictive for patients with CHL and least predictive for patients with mixed hearing loss with 4PTA BC ≥ 40 dB HL. For the test device at softband, results tended to show the minimum achievable outcome and the mEV tended to predict the realistically achievable outcome.

**Conclusion:**

Osia can be used for the treatment of CHL and MHL within the indication limits. The average preoperative bone conduction hearing threshold also provides an approximate estimate of the postoperative EV with Osia, for which the most accurate prediction is obtained using the preoperative mEV. Prediction accuracy decreases from a BC-4PTA of ≥ 40 dB.

Das aktive transkutane, teilimplantierbare, osseointegrierte Knochenleitungssystem Cochlear™ Osia® (Fa. Cochlear, Sydney, Australien) ist seit April 2021 im deutschsprachigen Raum zugelassen. Das Osia ist für Patienten mit Schallleitungs- oder kombinierter Schwerhörigkeit (SL-SH/komb-SH) mit einem durchschnittlichen Knochenleitungshörverlust von maximal 55 dB HL in den Frequenzen 0,5; 1; 2 und 4 kHz oder bei einseitiger Taubheit („single-sided deafness“, SSD) indiziert [12]. Der Vorteil von transkutanen im Vergleich zu perkutanen Knochenleitungssystemen ist v. a. die Reduktion der Häufigkeit von Hautentzündungen und Weichgewebereaktionen im Implantatbereich. Zusätzlich ist der kosmetische Aspekt nicht zu vernachlässigen. Insbesondere bei Personen mit einer Kurzhaarfrisur kann das perkutane System aus ästhetischen Gründen aufgrund der Sichtbarkeit nachteilig sein. Für Kinder ist eine transkutane Lösung im Hinblick auf Schwimmunterricht und sportliche Freizeitaktivitäten zu bevorzugen. Beide Systeme umgehen die gestörte Schallübertragung vom Mittelohr zum Innenohr. Somit ist, neben der medizinischen Indikation, die Beurteilung der audiologischen Indikation anhand der Knochenleitungshörschwelle von zentraler Bedeutung [13]. Ein weiteres Kriterium für die Indikationsstellung bzw. zur Abschätzung des zu erwartenden Versorgungserfolgs ist der maximale Ausgangspegel („maximum power output“, MPO) des Knochenleitungssystems [13, 14]. Der MPO stellt den frequenzabhängigen Verlauf der Maximalleistung als Schalldruckpegel (dB SPL) oder Kraftpegel (dB FL) dar.

Mit einem Knochenleitungssystem versorgte Patienten sollten einen Dynamikbereich von 30–35 dB zur Verfügung haben, um ausreichend Sprache verstehen zu können [13]. Wenn man diesen geforderten Dynamikbereich bei der Indikationsstellung berücksichtigt, reduziert sich die maximal mögliche durchschnittliche versorgbare Knochenleitungshörschwelle auf 40 dB HL (35 dB Dynamikbereich) bzw. 45 dB HL (30 dB Dynamikbereich) [13]. Aufgrund des steileren Lautheitswachstums kann bei Knochenleitungsimplantaten der minimal notwendige Zieldynamikbereich möglichweise auf 30 dB reduziert werden [16]. Für die Abschätzung des Behandlungserfolgs mit Knochenleitungssystemen vor Implantation besteht der Vorteil, dass diese Systeme durch Tragen am Softband getestet werden können. Jedoch muss die insbesondere im hochfrequenten Bereich ab 2 kHz vorhandene Dämpfung durch die Haut und Haare bei der Anpassung des Testsystems berücksichtigt werden, die ansonsten zu einer Reduktion des Sprachverstehens von etwa 10 Prozentpunkten bei 65 dB SPL führt [3]. Eine der geforderten Bedingungen für einen ausreichenden Nutzen einer Hörgeräteversorgung in Deutschland ist eine 20%ige Verbesserung des Sprachverstehens mit Hörhilfe bei 65 dB SPL [17]. Wenn jedoch das unversorgte Sprachverstehen bei 65 dB SPL nicht messbar ist, ist das maximale Einsilberverstehen (mEV) heranzuziehen, welches als prognostischer Wert für das postoperative Einsilberverstehen angenommen werden kann [8, 9].

Das Ziel dieser retrospektiven Studie ist daher die Analyse der Vorhersagbarkeit des postoperativen EV bei 65 dB SPL mit dem Knochenleitungssystem Osia anhand der präoperativ vorhandenen audiometrischen Daten (Knochenleitungshörschwelle, unversorgtes mEV bzw. des EV bei 65 dB SPL mit Knochenleitungstestgerät am Softband). Darüber hinaus wird in dieser Arbeit untersucht, ob sich das mit Osia erreichte Sprachverstehen von Patienten mit reiner SL-SH von denen mit komb-SH unterscheidet und ob die Schallleitungskomponente (Abstand zwischen Luft- und Knochenleitung, „air-bone gap“, ABG)) einen Einfluss auf das Ergebnis aufweist. Hierfür erfolgt eine Analyse der Ergebnisse derjenigen Patienten, die anhand der über 4 Frequenzen gemittelten Hörschwelle in Knochenleitung („four-frequency pure-tone average“, PTA4-KL) den geforderten Dynamikbereich von 30–35 dB nicht erreichen, also eine durchschnittliche KL-Schwelle über 40 bzw. 45 dB HL aufweisen.

## Studiendesign und Untersuchungsmethoden

Die vorliegende Studie wurde mit Zustimmung der Ethik-Kommission der Universität Freiburg (Nr. 21/1142) im Einklang mit nationalem Recht sowie gemäß der Deklaration von Helsinki von 2013 (in der aktuellen, überarbeiteten Fassung) durchgeführt (Deutsches Register Klinischer Studien, DRKS 00024640).

### Patienten

In eine retrospektive Untersuchung wurden 29 erwachsene Patienten (36 Ohren) eingeschlossen, die zwischen 2017 und 2022 mit einem Osia-System (OSI100 oder OSI200) versorgt wurden. Von diesen Patienten nahmen 9 (11 Ohren) bereits an der multizentrischen Zulassungsstudie CBAS5539 teil [10]. Weitere 8 Patienten (12 Ohren) wurden in die retrospektive Langzeitanalyse nach Osia-Implantation aufgenommen, welche 2022 publiziert wurde [15]. Die demografischen und die audiometrischen Daten der Patienten sind in Tab. 1 aufgelistet.

**Tab. 1:** Demografische und audiologische Patientendaten

Demografische Daten						Audiologische Daten									
						Präoperativ unversorgt						Präoperativ KL-Testgerät		Postoperativ Osia	
ID	Geschlecht	Alter (LJ)	Frühere Studienteilnahme	Ätiologie	Seite	Art der Hörstörung	PTA4-KL (dB HL) ipsilateral	PTA4-LL (dB HL) ipsilateral	PTA4-KL (dB HL) kontralateral	PTA4-LL (dB HL) kontralateral	mEV ipsilateral (%)	Testgerät	FBE@65 dB mit Testgerät (%)	FBE@65 dB mit Osia (%)	Follow-up (Monate)
1	M	37	1;2	Cholesteatom bds., Radikalhöhle re	Rechts links	SL-SH	18,7 12,5	61,2 33,7	–	–	100 100	Baha 5 Power Baha 5 Power	85 65	95 100	61
2	M	33	1;2	COM	Links	komb-SH‑I	25.0	60,0	Kongenitale Surditas		90	Baha BP110	85	90	61
3	M	27	1;2	GG-Atresie	Rechts	SL-SH	8,7	62,5	5,0	6,0	95	Baha BP110	90	90	61
4	W	61	1;2	Tympanosklerose	Rechts	komb-SH-II	40,0	86,2	40,0	41,0	70	Baha BP110	30	80	61
5	M	18	1;2	GG-Atresie (Nager-Syndrom)	Rechts links	komb-SH‑I	27,5 26,2	65,0 65,0	–	–	95 95	Baha BP110 Baha BP110	80 75	100 100	61
6	M	52	1;2	Cholesteatom	Rechts	SL-SH	17,5	45,5	22,5	25,0	100	Baha BP110	80	95	58
7	W	77	1;2	Radikalhöhle	Rechts	komb-SH-II	47,5	92,5			65	Ponto 3 SP	45	60	58
8	W	30	1;2	Radikalhöhle	Rechts	komb-SH‑I	35,0	105,0	15,0	16,5	70	Baha 5 Power	50	85	58
9	M	39	1;2	Radikalhöhle	Links	SL-SH	8,7	66,7	6,0	7,7	95	Baha BP110	100	95	58
10	M	58	2	Radikalhöhle re, GG-Stenose li, Z. n. Baha li	Rechts links	komb-SH-II	41,2 45,0	69,2 55,6	–	–	90 90	Baha BP110 Baha BP110	50 70	65 80	50
11	W	64	2	GG-Entzündung	Rechts	komb-SH‑I	30,0	70,5	21,5	30,7	70	Baha 5 Power	65	70	50
12	M	40	2	Radikalhöhle re, Z. n. Baha bds.	Rechts links	komb-SH‑I	23,7 23,7	45,5 43,7	–	–	100 100	Baha 5 Power Baha 5 Power	80 80	95 95	51
13	W	43	2	Otosklerose	Links	komb-SH-II	42,5	74,2	31,2	32,7	85	Baha 5 Power	35	80	45
14	W	69	2	Radikalhöhle	Links	SL-SH	18,7	51,75	18,0	20,7	95	Baha 5	95	90	39
15	M	52	2	Glomustumor, GG-Obliteration	Links	komb-SH‑I	22,5	77,5	21,2	25,0	80	Ponto 3 SP	80	80	39
16	W	57	2	Cholesteatom, Radikalhöhle bds.	Rechts links	komb-SH‑I	20,0 28,7	55,2 47,5	–	–	100 95	Ponto 3 SP Ponto 3 SP	85 85	95 95	38
17	M	59	2	GG-Entzündung bds.	Rechts links	komb-SH‑I	31,2 33,7	55,0 88,7	–	–	95 80	Ponto 3 SP Ponto 3 SP	95 90	90 90	28
18	W	58	–	Radikalhöhle	Rechts	komb-SH‑I	26,2	60,5	19,5	27,0	100	Baha 5 Power	70	90	20
19	W	53	–	Cholesteatom, Radikalhöhle bds.	Rechts links	komb-SH‑I	35,0 30,0	53,5 41,2	–	–	100 100	Ponto 3 SP Ponto 3 SP	60 65	95 95	20
20	M	77	–	Radikalhöhle	Links	SL-SH	18,7	35	20,0	26,0	100	Baha 5	90	95	18
21	M	54	–	Cholesteatom	Rechts	SL-SH	15,0	39,5	Surditas li, CI li		100	Baha 5	75	95	15
22	W	27	–	COM	Rechts	SL-SH	10,5	47,5	11,2	26,7	100	Baha 5 Power	90	95	13
23	M	55	–	GG-Stenose	Links	komb-SH‑I	37,5	66,2	26,7	46,7	80	Baha 5 SP	60	85	11
24	M	60	–	GG-Stenose	Rechts	komb-SH‑I	27,5	63	25,7	28,7	85	Baha 5 Power	60	80	6
25	W	36	–	Granulomatose mit Polyangiitis	Links	SL-SH	16,2	66,5	11,2	12,5	90	Baha 5 SP	65	75	5
26	M	46	–	Radikalhöhle	Links	komb-SH‑I	27,5	72,0	17,7	21,2	85	Ponto 3 SP	70	90	4
27	M	46	–	Cholesteatom	Links	komb-SH‑I	33,7	78,0	28,7	30,5	75	Baha 5 Power	40	75	4
28	W	60	–	Tympanosklerose	Links	komb-SH‑I	28,7	68,0	20,0	22,5	90	Ponto 3 SP	95	100	4
29	W	23	–	COM	Links	SL-SH	13,7	48,2	12,0	17,0	100	Baha 6 Max	100	95	1

*1*: Mylanus et al. (2020) [10], *2*: Rauch et al. (2022) [15], *Baha *knochenverankertes Hörgerät („bone-anchored hearing aid“),* BEV@65* *dB* Freiburger Einsilbertest bei 65 dB SPL, *COM* chronische Otitis media, *GG* Gehörgang, *komb-SH‑I* kombinierte Schwerhörigkeit mit PTA4-KL ≥ 20 dB HL und < 40 dB HL, *komb-SH-II* kombinierte Schwerhörigkeit mit PTA4-KL ≥ 40 dB HL, *LJ* Lebensjahre, *M* männlich, *mEV* maximales Einsilberverstehen bei jedem SPL möglich, *Seite* bezieht sich auf die Implantatseite(n), *SL-SH* Schallleitungsschwerhörigkeit mit PTA4-KL < 20 dB HL, *SP* SuperPower,* W* weiblich

### Methoden

#### Audiologische Messungen und Geräteprogrammierung

Die Daten wurden im Rahmen der klinischen Routine für die Leistungsbewertung des Osia-Implantats erhoben. Geräteprogrammierung und audiologische Messungen wurden in einem schallisolierten Raum (DIN EN ISO 8253) durchgeführt. Präoperativ wurden, je nach Patient, die Soundprozessoren Baha BP110, Baha 5, Baha 5 SP und Baha 5 Power (Fa. Cochlear) und der Soundprozessor Ponto 3 (Fa. Oticon Medical, Kopenhagen, Dänemark) als Testgeräte eingesetzt (Tab. 1). Bei der Anpassung der Soundprozessoren am Softband wurde das in der Anpasssoftware implementierte präskriptive schwellenbasierte Verfahren verwendet. Dabei wird der frequenzspezifische Verstärkungsbedarf anhand der mit dem Soundprozessor am Softband in situ gemessenen Hörschwellen festgelegt. Nach dieser Voreinstellung erfolgte eine Feinanpassung und im Fall von Rückkopplungen eine Reduktion der Verstärkung. Die postoperative Anpassung des Osia-Soundprozessors beinhaltete ebenfalls die präskriptive Festlegung des frequenzspezifischen Verstärkungsbedarfs anhand der in situ gemessenen Hörschwellen, eine Feinanpassung und eine Verstärkungsreduktion im Rückkopplungsfall.

#### Hörschwellenbestimmung

Prä- und postoperativ wurden die unversorgten Schwellen in Knochenleitung (KL) und Luftleitung (LL) bei den Frequenzen 0,25; 0,5; 0,75; 1; 2; 3; 4; 6 und 8 kHz mit Kopfhörern gemessen, wobei das kontralaterale Ohr mit Schmalbandrauschen maskiert wurde. Bei allen Patienten wurde die über die 4 Frequenzen 0,5; 1; 2 und 4 kHz gemittelte Hörschwelle („four-frequency pure-tone average“, PTA4) in KL und LL präoperativ bestimmt.

#### Sprachverstehen in Ruhe

Präoperativ wurde das unversorgte Sprachverstehen in Ruhe mithilfe des Freiburger Einsilbertests mittels Kopfhörer (Beyerdynamic DT48 oder Sennheiser HDA300) bei verschiedenen Schallpegeln und das mEV bestimmt. Dabei wurde eine Liste pro Pegel verwendet. Der maximale Darbietungspegel betrug 120 dB SPL. Präoperativ mit Testgerät am Softband sowie postoperativ mit Osia erfolgten die Messungen des Einsilberverstehens bei 65 dB SPL bei Präsentation der Sprache von vorn. Zur Maskierung des kontralateralen Ohrs wurde Breitbandrauschen mit 70 dB SPL über Kopfhörer präsentiert. Da es sich um eine retrospektive Auswertung handelt, erhielten die Patienten aus organisatorischen Gründen und je nach individuellen Präferenzen unterschiedliche, an die jeweilige PTA4-KL angepasste Testgeräte zur Erprobung am Softband (Tab. 1).

### Statistische Analyse

Nach Testung auf Normalverteilung wurden Gruppenvergleiche anhand Mittelwertvergleichen mittels einfaktorieller Varianzanalyse („analysis of variance“, ANOVA) und Post-hoc-Tests durchgeführt. Entsprechend der Testung auf Varianzgleichheit wurden bei gleichen Varianzen eine ANOVA und Tukey-post-hoc-Tests und bei ungleichen Varianzen eine Welch-ANOVA und Games-Howell-post-hoc-Tests verwendet. Das Signifikanzniveau war 0,05.

Für mehrfache Tests wurde nach Bonferroni korrigiert.

Für die Berechnung der Vorhersagegenauigkeit des postoperativen Sprachverstehens anhand jeder der beiden präoperativen EV-Größen sowie der präoperativen PTA4-KL wurde eine einfache lineare Regression verwendet und die Anpassungsgüte bzw. Varianzaufklärung gemäß der Klassifikation nach Cohen 1988 beurteilt. Für die Angabe der Varianzaufklärung *R*^*2*^ wurde jeweils das korrigierte *R*^*2*^ zur Vermeidung eines überschätzten Effekts verwendet.

Der grafische Zusammenhang zwischen der präoperativen PTA4-KL und dem präoperativen mEV bzw. dem postoperativen Einsilberverstehen wurde jeweils mittels logistischer Regression gemäß der unten aufgeführten Gleichung ermittelt. Hierbei kam das das Newton-Raphson-Verfahren zur Anwendung.

1 $$EV\left[{\%}\right]=100\frac{e^{\left(\beta _{0}+\beta _{1}PTA4\right)}}{1+e^{\left(\beta _{0}+\beta _{1}PTA4\right)}}$$

Die Varianzanalysen und zugehörigen Post-hoc-Tests sowie die linearen Regressionen erfolgten mittels SPSS (Version 27, Fa. IBM, Armonk, NY, USA) und die nichtlinearen Regressionen mittels Matlab (Version 9.7, Fa. Mathworks, Natick, MA, USA).

Unterschiedliche Ergebnisse des Freiburger Einsilbertests wurden hinsichtlich des Signifikanzniveaus nach Winkler und Holube [20] bewertet.

## Ergebnisse

Von den 29 in die Studie eingeschlossenen Patienten wurden 7 beidseitig implantatversorgt (insgesamt 36 Ohren). Entsprechend der mittleren KL-Hörschwelle wurden die Patienten in 3 Gruppen unterteilt: (1) SL-SH, (2A) komb-SH‑I, (2B) komb-SH-II. Von den 29 Patienten wiesen 10 Patienten (11 Ohren) eine PTA4-KL < 20 dB HL und somit eine SL-SH (1) auf. Bei 19 Patienten (25 Ohren) bestand eine PTA4-KL ≥ 20 dB HL und damit eine komb-SH (2). Die Patienten mit komb-SH wurden wiederum in eine Gruppe mit 20 dB HL ≤ PTA4-KL < 40 dB HL (2A) unterteilt (komb-SH‑I, *n* = 15 Patienten, 20 Ohren) vs. PTA4-KL von ≥ 40 dB HL (2B, komb-SH-II, *n* = 4 Patienten, 5 Ohren). Außerdem wurden die Patienten entsprechend ihrer SL-Komponente in weitere 3 Gruppen differenziert, welche in allen Abbildungen durch Farbmarkierungen erkennbar sind (grün: *n* = 5, ABG < 20 dB; blau: *n* = 19, 20 dB ≤ ABG < 40 dB; rot: *n* = 12, ABG ≥ 40 dB).

### Präoperative audiometrische Ergebnisse

Der durchschnittliche präoperative PTA4-Wert auf der implantatversorgten Seite betrug für alle Patienten 26,4 ± 10,3 dB HL in Knochenleitung und 61,6 ± 16,3 dB HL in Luftleitung.

Die durchschnittlichen Hörschwellen des kontralateralen Ohrs betrugen 23,8 ± 9,8 dB HL (Knochenleitung) und 37,8 ± 19,3 dB HL (Luftleitung).

**Abb. 1:**
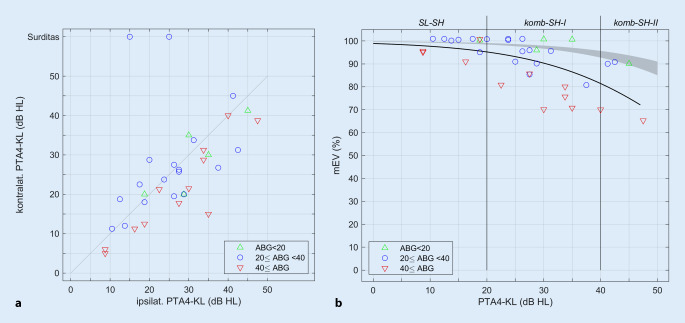
**a** Zusammenhang zwischen ipsi- und kontralateraler gemittelter Knochenleitungshörschwelle (*PTA4-KL*). *Werte oberhalb der Winkelhalbierenden* schlechtere kontralaterale Knochenleitungsschwelle. **b** Über Luftleitung gemessenes, präoperatives maximales Einsilberverstehen (*mEV*) in Abhängigkeit von der PTA4-KL. Schallleitungskomponente („air-bone gap“, *ABG*) anhand *verschiedenfarbiger Symbole* dargestellt. *Grauer Bereich* mittleres mEV in Abhängigkeit vom Innenohrhörverlust unter Berücksichtigung der 95%-Konfidenzintervalle für die Parameter einer logistischen Regression [4], *schwarze Linie* mittleres mEV, logistische Regression nach Gl. 1, *komb-SH‑I* kombinierte Schwerhörigkeit (Gruppe I), *komb-SH-II* kombinierte Schwerhörigkeit (Gruppe II), *SL-SH* Schallleitungsschwerhörigkeit

Die Abb. 1a zeigt die Relation von ipsi- und kontralateralen Knochenleitungshörschwellen in Bezug auf die Seite der Osia-Implantation. Die mittlere Seitendifferenz der PTA4-KL lag bei 6 dB. In 11 der 12 Fälle mit einem ipsilateralen ABG ≥ 40 dB fand sich eine im Vergleich zum ipsilateralen Ohr geringere PTA4-KL des kontralateralen Ohrs.

Die mittleren Seitendifferenzen für die beiden Gruppen mit geringerer SL-Komponente lagen, ohne Berücksichtigung der beiden Fälle mit kontralateraler Surditas, unterhalb von 2 dB.

Die SL-Komponente war zwischen den 3 Gruppen nicht signifikant verschieden (Tab. 2). Das präoperative mEV unterschied sich signifikant zwischen den Gruppen SL-SH und komb-SH‑I (Tab. 2). Es bestanden keine signifikanten Unterschiede im präoperativen mEV zwischen den Gruppen SL-SH und komb-SH-II sowie zwischen komb-SH‑I und –II (Tab. 2). Das präoperative EV mit KL-Testgerät war für SL-SH signifikant besser als für komb-SH-II (Tab. 2). Zudem war das präoperative EV mit KL-Testgerät für komb-SH‑I signifikant besser als für komb-SH-II (Tab. 2).

**Tab. 2:** Übersicht der Ergebnisse der Varianzanalysen und Post-hoc-Mittelwertvergleiche der Hörschwellen im mit einem Implantat zu versorgenden bzw. im implantatversorgten Ohr zwischen den 3 Gruppen

Untersuchte Variable	ANOVA (*p*-Wert)	Gruppenvergleich: *p*-Wert und Mittelwert ± Standardabweichung (dB HL)		
		*SL-SH vs.* *Komb-SH‑I*	*SL-SH vs. komb-SH-II*	*komb-SH‑I vs. Komb-SH-II*
*Präoperative PTA4-KL*	*p* < 0,001	***p*** **<** 0,001 (***)^a^	***p*** **<** 0,001 (***)^a^	***p*** **<** 0,001 (***)^a^
		14,48 ± 3,91 vs. 28,69 ± 4,65	14,48 ± 3,91 vs. 43,25 ± 3,01	28,69 ± 4,65 vs. 43,25 ± 3,01
*Präoperative PTA4-LL*	*p* = 0,008	*p* = 0,051 (ns)^a^	***p*** **=** 0,009 (**)^a^	*p* = 0,265 (ns)^a^
		50,75 ± 12,09 vs. 64,06 ± 15,63	50,75 ± 12,09 vs. 75,57 ± 14,50	64,06 ± 15,63 vs. 75,57 ± 14,50
*Präoperative SL-Komponente*	*p* = 0,873	*p* = 0,984 (ns)^a^	*p* = 0,864 (ns)^a^	*p* = 0,903 (ns)^a^
		36,27 ± 13,64 vs. 35,38 ± 14,39	36,27 ± 13,64 vs. 32,32 ± 14,54	35,38 ± 14,39 vs. 32,32 ± 14,54
*Präoperatives mEV*	*p* = 0,005	***p*** **=** 0,007 (**)^b^	*p* = 0,081 (ns)^b^	*p* = 0,418 (ns)^b^
		97,73 ± 3,44 vs. 89,25 ± 10,42	97,73 ± 3,44 vs. 81,00 ± 12,45	89,25 ± 10,42 vs. 81,00 ± 12,45
*Präoperatives EV bei 65* *dB SPL mit KL-Testgerät*	*p* < 0,001	*p* = 0,085 (ns)^a^	***p*** **<** 0,001(***)^a^	***p*** **=** 0,002 (**)^a^
		85,00 ± 12,45 vs. 73,00 ± 15,25	85,00 ± 12,45 vs. 46,00 ± 15,57	73,00 ± 15,25 vs. 46,00 ± 15,57
*Postoperatives EV bei 65* *dB SPL mit Osia*	*p* < 0,001	0,65 (ns)^a^	***p*** **<** 0,001 (***)^a^	***p*** **<** 0,001 (***)^a^
		92,73 ± 6,47 vs. 89,50 ± 8,26	92,73 ± 6,47 vs. 71,00 ± 8,94	89,50 ± 8,26 vs. 71,00 ± 8,94

*ANOVA* Varianzanalyse („analysis of variance“), *KL* Knochenleitung, *komb-SH‑I* kombinierte Schwerhörigkeit (Gruppe I), *komb-SH-II* kombinierte Schwerhörigkeit (Gruppe II), *LL* Luftleitung, *(m)EV* (maximales) Einsilberverstehen,* ns *nicht signifikant,* PTA4* „four-frequency pure tone average“, *SL-SH* Schallleitungsschwerhörigkeit

*Fettdruck *(hoch)signifikante *p*-Werte

^a^Tukey-post-hoc-Tests

^b^Games-Howell-post-hoc-Tests

### Darstellung des präoperativen mEV in Abhängigkeit von der gemittelten KL-Schwelle 

Darstellung des präoperativen mEV in Abhängigkeit von der gemittelten KL-Schwelle gestaltete sich folgendermaßen: In Abb. 1b ist das präoperativ gemessene mEV in Abhängigkeit von der gemittelten Knochenleitungshörschwelle (PTA4-KL) dargestellt. Für Knochenleitungshörschwellen bis zu 45 dB HL lag das mEV zwischen 65 und 100 %. Der PTA4-KL-Wert erklärte 37 % der gefundenen Variabilität (R_Spearman_ = 0,61; *p* < 0,001). Fälle mit einem ABG > 40 dB HL lagen signifikant unter dem als schwarze Linie dargestellten mittleren mEV in Abhängigkeit vom PTA4-KL-Wert (Vorzeichentest, *p* = 0,036). Alle Fälle mit einem ABG < 20 dB HL wiesen ein mEV oberhalb dieses Mittelwerts auf.

Die logistische Regression nach Gl. 1 ergibt β_0_ = 4,50 ± 0,21 und β_1_ = −0,0756 ± 0,0061. Der Vergleich mit dem mittleren mEV einer Patientengruppe mit rein sensorineuraler Schwerhörigkeit [5] zeigt, dass die in der vorliegenden Studie untersuchten Patienten aufgrund der Messung des mEV über Luftleitung hier signifikant geringere Werte in Abhängigkeit vom Ausmaß der Innenohrschwerhörigkeit aufweisen. Seinerzeit [4] wurden Werte von β_0_ = 5,99 ± 0,08 und β_1_ = −0,0756 ± 0,0012 festgestellt. Die in dieser Studie ermittelten größeren Konfidenzintervalle für die Parameter β sind durch die geringeren Fallzahlen bedingt.

### Postoperative audiometrische Ergebnisse

**Abb. 2:**
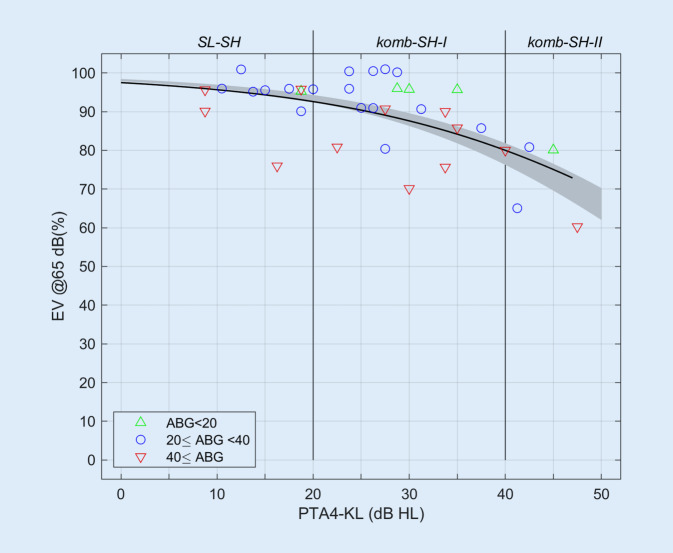
Das im Freifeld bei 65 dB SPL mit Osia erzielte postoperative Einsilberverstehen (*EV*) in Abhängigkeit von der gemittelten Knochenleitungshörschwelle (*PTA4-KL*). Unterschiedliche Bereiche der Schallleitungskomponente („air-bone gap“, *ABG*) mittels *verschiedenfarbiger Symbole* dargestellt. *Grauer Bereich* mittleres EV mit Luftleitungshörgerät in Abhängigkeit vom Innenohrhörverlust unter Berücksichtigung der 95%-Konfidenzintervalle für die Parameter einer logistischen Regression [5], *schwarze Linie* mittleres EV mit Osia, logistische Regression nach Gl. 1, *komb-SH‑I* kombinierte Schwerhörigkeit (Gruppe I), *komb-SH-II* kombinierte Schwerhörigkeit (Gruppe II), *SL-SH* Schallleitungsschwerhörigkeit

Die Abb. 2 zeigt das postoperativ gemessene EV in Abhängigkeit von der gemittelten Knochenleitungshörschwelle. Im Bereich von Knochenleitungshörschwellen bis zu 45 dB HL lag das postoperativ erreichte EV zwischen 60 und 100 %. Der PTA4-KL-Wert erklärte jedoch nur 25 % der festgestellten Variabilität des EV (R_Spearman_ = −0,51; *p* = 0,0016). In den Fällen mit einer ABG > 40 dB HL lag das EV signifikant unter dem als schwarze Linie dargestellten mittleren EV (Vorzeichentest, *p* = 0,036). In 4 von 5 Fällen mit einer ABG < 20 dB HL war das EV größer als das mittlere EV.

Die logistische Regression nach Gl. 1 ergibt β_0_ = 3,67 ± 0,17 und β_1_ = −0,0570 ± 0,0054. Der Vergleich mit einer Gruppe von Hörgeräteträgern mit ausschließlich sensorineuraler Schwerhörigkeit aus einer vorangegangenen Studie [5], grauer Bereich, zeigt, dass die mittleren Versorgungsergebnisse mit Osia im Bereich des 95%-Konfidenzintervalls für Hörgeräteträger liegen. Dort [4] wurden die Parameter zu β_0_ = 3,98 ± 0,05 und β_1_ = −0,0661 ± 0,0008 bestimmt.

### Vorhersage der postoperativen audiometrischen Ergebnisse mit Osia

#### Vorhersage anhand der präoperativen PTA4-KL

In der Zusammenfassung der Ergebnisse aller Gruppen konnte die präoperative PTA4-KL das postoperative EV bei 65 dB SPL mit Osia mit einer Varianzaufklärung von 31 % vorhersagen (*R*^*2*^ = 0,310; *F*(1,34) = 16,709, *p* < 0,001; *β* = −0,570, *p* < 0,001). Die lineare Regression für jede der einzelnen Gruppen war nicht signifikant (jeweils *p (ANOVA)* ≥ 0,05). Die Linearität der Zusammenhänge wurde mittels Streu‑/Punktediagramm vorab überprüft. Bei der Gruppe komb-SH-II war der lineare Zusammenhang aufgrund des geringen *n* nicht überprüfbar.

#### Vorhersage anhand des präoperativen EV mit KL-Testsystem

Bei Berücksichtigung aller Gruppen war eine Vorhersage des postoperativen EV mit Osia anhand des präoperativen EV mit KL-Testgerät bei 65 dB SPL mit einer Varianzaufklärung von 36,3 % möglich (*R*^*2*^ = 0,363; *F*(1,34) = 20,957, *p (ANOVA)* < 0,001; *β* = 0,338, *p* < 0,001). Bei separater Betrachtung der einzelnen Gruppen konnte eine signifikante Vorhersage nur für die Gruppe der komb. SH‑I mit einer Varianzaufklärung von 22,2 % gezeigt werden (*R*^*2*^ = 0,222; *F*(1,18) = 6,414, *p (ANOVA)* = 0,021; *β* = 0,288, *p* < 0,001, für SL und komb. SH-II jeweils *p (ANOVA)* ≥ 0,05).

#### Vorhersage anhand des präoperativen mEV

Das präoperative mEV aller Patienten konnte das postoperative EV bei 65 dB SPL mit Osia mit einer Varianzaufklärung von 53 % in signifikanter Weise vorhersagen (*R*^*2*^ = 0,53; *F*(1,34) = 40,497, *p (ANOVA)* < 0,001; *β* = 0,716, *p* < 0,001).

Für jede der beiden Gruppen SL-SH und komb-SH‑I ließ sich das postoperative EV mit Osia anhand des präoperativen mEV mit hoher Varianzaufklärung von 72,8 % für die Gruppe SL-SH (*R*^*2*^ = 0,728; *F*(1,9) = 27,753, *p (ANOVA)* < 0,001; *β* = 1,635, *p* < 0,001) und hoher Varianzaufklärung von 61 % für die Gruppe komb-SH‑I (*R*^*2*^ = 0,61; *F* (1,18) = 30,752, *p (ANOVA)* < 0,001; *β* = 0,654, *p* < 0,001) in signifikanter Weise vorhersagen. Für komb-SH-II war keine Vorhersage anhand des präoperativen mEV möglich (*p (ANOVA)* ≥ 0,05).

### Darstellung des postoperativen Einsilberverstehens

**Abb. 3:**
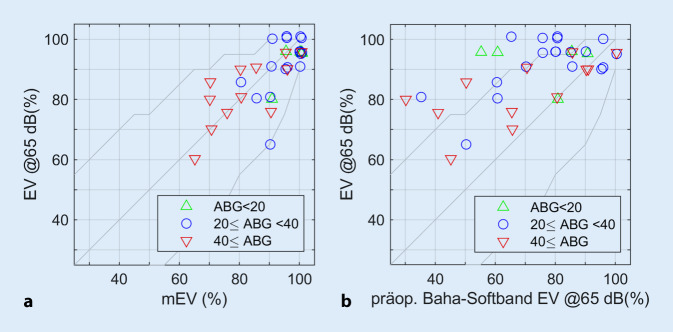
Zusammenhänge zwischen prä- und postoperativem Einsilberverstehen. **a** Mit dem Osia erreichtes Einsilberverstehen (*EV*) über dem maximalen Einsilberverstehen (*mEV*); **b** Relation zwischen EV und präoperativem EV mit dem KL-Testgerät am Softband. Bereiche der Schallleitungskomponente („air-bone gap“, *ABG*) über *verschiedenfarbige Symbole* dargestellt. *Baha* knochenverankertes Hörgerät („bone-anchored hearing aid“)

Die Darstellung des postoperativen Einsilberverstehens mit Osia wies eine Abhängigkeit vom präoperativ gewählten Test auf. In Abb. 3 sind die Beziehungen zwischen präoperativen sprachaudiometrischen Befunden, mEV (Abb. 3a) bzw. EV mit dem KL-Testgerät am Softband (Abb. 3b), und dem postoperativen EV mit Osia dargestellt. Unter Berücksichtigung der geringen Patientenzahl zeigt der Vergleich beider Teilabbildungen eine Tendenz, dass das präoperative mEV näher am erreichbaren Versorgungsergebnis liegt als das EV bei präoperativer Testung mit einem KL-Testgerät am Softband.

Das im Mittel bei 91 % liegende präoperative mEV wurde in 34 der 36 Fälle (94 %) im Rahmen der Genauigkeit des Freiburger Einsilbertests [20] erreicht bzw. übertroffen. Das präoperativ mit dem KL-Testgerät erzielte EV von im Mittel 73 % wurde mit Osia in allen Fällen erreicht bzw. übertroffen. In keinem der Fälle wurde bei der präoperativen Testung mit dem Softband ein höheres EV als das mEV beobachtet. Insgesamt unterscheidet sich das mittlere mEV (94 %) weniger vom mittleren postoperativen EV mit Osia (88 %) als das präoperativ mit dem KL-Testsystem (bei 65 dB SPL) erreichte EV.

## Diskussion

Die dargestellten Ergebnisse bestätigen, dass das Knochenleitungssystem Osia eine erfolgreiche und sichere Alternative zu den perkutanen Knochenleitungssystemen darstellt, wie bereits von anderen Autoren berichtet wurde [1, 10, 15].

Ein Ziel dieser retrospektiven Studie war die Untersuchung der Vorhersagbarkeit der postoperativen Ergebnisse des Sprachverstehens mit dem Osia, um einerseits eine korrekte Indikationsstellung zur Implantation inklusive der Abgrenzung zu alternativen Implantaten treffen sowie die Patienten adäquat über das potenziell erreichbare Ergebnis mit dem Osia beraten zu können.

In der vorliegenden Studie konnte gezeigt werden, dass die Patienten nach der Osia-Versorgung ein Einsilberverstehen erreichen, welches mit den aktuellen Daten aus der Hörgeräteversorgung von Patienten mit ausschließlich sensorineuraler Schwerhörigkeit übereinstimmt [2, 4]. Aufgrund der Signaldarbietung über Luftschall für das mEV weisen Patienten mit kombinierter Schwerhörigkeit, insbesondere solche mit höherer Schallleitungskomponente, zwar präoperativ ein geringeres mEV auf, konnten dieses Potenzial im Unterschied zu konventionellen Hörgeräteträgern jedoch nahezu vollständig umsetzen. Inwieweit die Pegelbegrenzung bei der präoperativen mEV-Bestimmung hier einen Einfluss hat, bleibt aufgrund des retrospektiven Designs ungeklärt.

Die präoperative Abschätzbarkeit des Versorgungserfolgs anhand des präoperativ erfassten maximalen Einsilberverstehens wurde für Patienten mit Innenohrschwerhörigkeit ohne ABG von Hoppe et al. mehrfach belegt [4–6]. Für Patienten mit SL-SH oder komb-SH fanden sich lediglich 2 Arbeiten, welche die Ergebnisse nach Implantation von aktiven Mittelohrimplantaten und „direct acoustic cochlear stimulators“ (DACS) untersuchten [8, 9]. Müller et al. (2017) zeigten, dass das mit aktiven Mittelohrimplantaten erzielte postoperative EV bei 65 dB SPL vorwiegend von der Ankopplungsqualität des „floating mass transducer“ abhängig ist. Es wurde jedoch kein Einfluss der SL-Komponente auf das postoperative Einsilberverstehen beschrieben. Die in den Daten der vorliegenden Arbeit festgestellte mittlere Abweichung zwischen dem mEV und dem EV mit Osia von 2 Prozentpunkten liegt unterhalb der beschriebenen Differenzen für andere implantierbare Hörsysteme [8, 9] und ist am ehesten auf die standardisierte Ankopplung und geringere Variabilität der operativen Vorgehensweise im Vergleich zu aktiven Mittelohrimplantaten zurückzuführen.

Mit dem präoperativen mEV ließ sich das postoperative Sprachverstehen mit Osia besser als mit dem präoperativen EV mit KL-Testgerät am Softband, d. h. mit höherer Varianzaufklärung, vorhersagen. Unter Berücksichtigung der Hautdämpfung bei der Übertragung der Vibration unter Verwendung des KL-Testgeräts am Softband zeigen diese Testergebnisse eher das minimal zu erwartende postoperative EV, während sich mit dem mEV das realistisch zu erwartende postoperative EV bei 65 dB SPL vorhersagen lässt und damit eine insgesamt höhere Abschätzung möglich wird, welche in der vorliegenden Untersuchung auch noch in 34 von 36 Fällen (94 %) erreicht bzw. übertroffen wird.

Patienten mit einem geringeren ABG wiesen im Vergleich zu Patienten mit einem höheren ABG ein verbessertes postoperatives EV mit Osia auf im Vergleich zum präoperativen mEV. So erreichten Patienten mit einer SL-Komponente von ≥ 40 dB HL sowohl präoperativ ein schlechteres unversorgtes mEV als auch postoperativ mit Osia ein signifikant schlechteres EV bei 65 dB SPL im Vergleich zu Patienten mit einer geringeren SL-Komponente (< 40 dB HL). Insbesondere die Fälle mit einem ABG oberhalb 40 dB weisen mehrheitlich ein besseres Gegenohr auf.

Aufgrund dieser dem Effekt sogar gegenläufigen Konstellation muss ein besseres Gegenohr als Grund für den beobachteten Effekt ausgeschlossen werden. Eine potenzielle Ursache könnte das „Mithören“ des Gegenohrs über die Luftleitung bei der Freifeldmessung sein, d. h., bei einer SL-Komponente < 20 dB wird das Sprachsignal sowohl über das Osia mit 65 dB SPL als auch direkt über Luftleitung mit < ~45 dB SPL (Normalhörende: 100 % EV bei 45 dB SPL) übertragen. Auch bei mittleren Schallleitungskomponenten ist eine kontralaterale Übertragung des Direktschalls, allerdings mit geringerem Pegel, vorhanden. Eine weitere mögliche Erklärung ist eine mögliche Deprivation der Hörbahn aufgrund einer länger bestehenden hochgradigen Schallleitungsschwerhörigkeit, insbesondere bei nicht nutzbaren konventionellen Hörgeräten vor der Osia-Versorgung, wie z. B. bei Patienten mit Gehörgangsatresien [7, 21]. Die vorliegenden Ergebnisse zeigen eine negative Abhängigkeit des Sprachverstehens mit Osia vom ABG und weichen damit von den Ergebnissen von Mylanus et al. (1998) ab [11]. Snik et al. (2005) geben an, dass Knochenleitungshörsysteme bei einer Schallleitungsschwerhörigkeit von > 30 dB HL den konventionellen Luftleitungshörgeräten (LL-HG) überlegen sind [19]. Jedoch sind die sich zwischen den Studien unterscheidenden Untersuchungsdesigns und Versorgungszeitpunkte unbedingt zu berücksichtigen. Mylanus et al. (1998) und Snik et al. (2005) beziehen sich auf die Analyse eines intraindividuellen Vergleichs von LL-HG und KL-Geräten vor mehr als 25 Jahren. In der vorliegenden Arbeit handelt es sich um aktuelle Versorgungsergebnisse und deren Vergleich mit mittleren Sprachverständlichkeiten aus anderen Studien/Kliniken in Patientenpopulationen mit rein sensorineuraler Schwerhörigkeit.

Eine detaillierte Abklärung der Ursachen für die in der vorliegenden Arbeit gezeigten schlechteren Ergebnisse von Osia-Patienten mit einem ABG > 40 dB ist im Rahmen dieser retrospektiven Studie nicht möglich. Die sich hier andeutenden Abhängigkeiten des postoperativen EV vom ABG lassen eine präoperative Abschätzung des Versorgungserfolgs erwarten. Eine statistische Bestätigung dieser Beobachtungen sollten jedoch in zukünftigen prospektiven Studien mit entsprechender Zusammenstellung der Studienpopulation (und größerer Anzahl *n* für die Gruppe der PTA4-KL ≥ 40 dB HL) erfolgen.

Mit dem präoperativen maximalen EV konnte das postoperative SV mit Osia mit hoher Varianzaufklärung besser als mit dem präoperativen EV mit KL-Testgerät am Softband vorhergesagt werden. Die Betrachtung der einzelnen Gruppen zeigte, dass sich das präoperative mEV als Prädiktor des postoperativen EV mit Osia für die Gruppen SL-SH und komb-SH‑I gut, jedoch nicht für die Gruppe mit komb-SH-II eignet. Das postoperative EV der Gruppe komb-SH-II weist mit Werten zwischen 60 und 80 % eine große Spanne auf. Die vorliegenden Ergebnisse zeigen eine Tendenz, dass insbesondere Patienten mit einer PTA4-KL ≥ 40 dB HL, welche somit einen limitierten Dynamikbereich von ≤ 30 dB aufweisen [14], ein EV mit Osia erreichen, welches sich nur wenig von dem maximalen EV der aktuellen Cochleaimplantat(CI)-Indikation von 60 % bei 65 dB SPL [18] unterscheidet. Somit sollten die firmenseitigen Indikationsgrenzen mit einem PTA4-KL-Wert von maximal 55 dB HL sehr kritisch betrachtet werden. Auch wenn in der vorliegenden Studie nur eine limitierte Patientenzahl mit einer PTA4-KL ≥ 40 dB vorgewiesen werden kann, zeigt sich in den Vorhersagemodellen der Autoren, dass die maximal mögliche durchschnittliche versorgbare Knochenleitungshörschwelle auf 45, eher 40 dB HL reduziert werden sollte, um ein ausreichendes Sprachverstehen zu erreichen und eine Toleranz bezüglich potenzieller Progredienz des Hörverlusts zu gewährleisten. In der vorliegenden Arbeit wurde die Vorhersagbarkeit des postoperativen Ergebnisses mit Osia in Abhängigkeit vom präoperativen PTA4-Wert und mEV untersucht. Vergleichbare Untersuchungen mit anderen Knochenleitungssystemen sind aktuell nicht publiziert. Eine prospektive Untersuchung bezüglich dieser Fragestellung und insbesondere zur Evaluation der Ergebnisse von Patienten im Grenzbereich der Indiktionen ist für alle Knochenleitungssysteme empfehlenswert.

Insbesondere für Patienten mit einem PTA4-KL-Wert ≥ 40 dB ist eine individuelle Entscheidung für das jeweilige implantierbare Hörsystem (perkutanes Superpower-KL-Implantat, aktives Mittelohrimplantat, CI) unter Berücksichtigung der Progredienz und der Ätiologie der Schwerhörigkeit, der anatomischen Befunde sowie v. a. des Alters, der Narkosefähigkeit und des Allgemeinzustands der Patienten notwendig und kritisch zu treffen.

## Fazit für die Praxis

- Das Osia-System kann für die Versorgung von Schallleitungs- und kombinierter Schwerhörigkeit mit einer durchschnittlichen versorgbaren Knochenleitungshörschwelle von maximal 45, eher 40 dB HL mit guter Erfolgsvorhersagbarkeit eingesetzt werden.
- Das präoperative Sprachverstehen mit KL-Testgerät am Softband kann dabei zur unteren Abschätzung des postoperativen Sprachverstehens mit Osia herangezogen werden.
- Sowohl die mittlere präoperative Knochenleitungshörschwelle als auch das präoperative maximale Einsilberverstehen (EV) erlauben eine Abschätzung des postoperativen EV, das sich anhand des präoperativen maximalen EV genauer vorhersagen lässt.
- Die vorliegende Schallleitungskomponente ist ebenso wie die Knochenleitungshörschwelle für das postoperativ erreichbare Sprachverstehen wesentlich.
- Die Abschätzung des postoperativen Versorgungserfolgs hat dabei für Patienten mit Schallleitungsschwerhörigkeit oder kombinierter Schwerhörigkeit mit einer Knochenleitungshörschwelle < 40 dB HL eine hohe Vorhersagegenauigkeit, während Patienten mit Knochenleitungshörschwelle von ≥ 40 dB HL über eine schlechtere Vorhersagbarkeit sowie das potenzielle Nichterreichen des präoperativen maximalen EV aufgeklärt werden sollten.
